# Mechanisms of immune-tolerance to allergens on the mucosal surfaces

**DOI:** 10.1186/2045-7022-1-S1-S1

**Published:** 2011-08-12

**Authors:** Cezmi Akdis

**Affiliations:** 1 Swiss Institute of Allergy & Asthma Research, Department of Immunology, Davos, Switzerland

## 

The immune system is a highly interactive network, which makes it’s decisions on the basis of all body tissues, infections, normal flora bacteria and almost any environmental agents. In recent years, regulatory T cells (TReg) cells have become a prime target for strategies aimed at inducing tolerance to ffod antigens. Immune tolerance in the context of allergy can be defined as persistence of efficacy following discontinuation of treatment, implying an altered allergen-specific memory T and B cell response. Various populations of TReg cells have been shown to play a central role and their identification as key regulators of immunological processes in peripheral tolerance to food- and aero-allergens has opened an important era in the prevention and treatment of allergic diseases. Both naturally occurring CD4+ CD25+ TReg cells and inducible populations of allergen-specific interleukin-10 (IL-10)-secreting T regulatory type 1 (Tr1) cells inhibit allergen-specific effector cells in experimental models. Skewing of allergen-specific effector T cells to a regulatory phenotype appears as a key event in the development of healthy immune response to allergens and successful outcome in allergen-specific immunotherapy. FoxP3+ CD4+CD25+ TReg cells and Tr1 cells contribute to the control of allergen-specific immune responses in several major ways, which can be summarized as suppression of dendritic cells that support the generation of effector T cells; suppression of effector Th1, Th2 and Th17 cells; suppression of allergen-specific IgE, induction of IgG4; suppression of mast cells, basophils and eosinophils; interaction with resident tissue cells and remodeling, suppression of effector T cell migration to tissues. Current strategies for drug development exploit these observations with the potential for preventive therapies and cure for allergic diseases.

